# Severe Case of Rickettsiosis Identified by Metagenomic Sequencing, China

**DOI:** 10.3201/eid2705.203265

**Published:** 2021-05

**Authors:** Zhongqiu Teng, Yan Shi, Yao Peng, Huayi Zhang, Xia Luo, Xinchang Lun, Lianxu Xia, Yuanhai You, Zhenpeng Li, Wen Zhang, Ying Zhang, Shicun Dong, Wentao Guo, Biao Kan, Bo Pang, Jianguo Xu, Aiping Qin

**Affiliations:** National Institute for Communicable Disease Control and Prevention, Chinese Center for Disease Control and Prevention, Beijing, China (Z. Teng, Y. Peng, X. Luo, X. Lun, L. Xia, Y. You, Z. Li, W. Zhang, B. Kan, B. Pang, J. Xu, A. Qin);; Qinghai Center for Disease Control and Prevention, Xining, China (Y. Shi, H. Zhang, S. Dong);; Baotou Medical College, Baotou, China (Y. Zhang);; Institute of Endemic Diseases of Qinghai, Xining (W. Guo)

**Keywords:** Rickettsiosis, *Rickettsia sibirica* subsp. *sibirica* BJ-90, Qinghai–Tibet Plateau, metagenomic sequencing, tickborne diseases, China, vector-borne infections, bacteria

## Abstract

A case of *Rickettsia sibirica* subspecies *sibirica* BJ-90 infection in China was identified by metagenomic analysis of an eschar biopsy specimen and confirmed by nested PCR. Seroprevalence of spotted fever group *Rickettsia* was ≈17.4% among the local population. This report highlights the threat of rickettsioses to public health in the Qinghai–Tibet Plateau.

*Rickettsia*, mainly transmitted by ticks, are a group of obligate gram-negative bacteria that cause mild to life-threatening rickettsioses. Two main groups of *Rickettsia* have been described on the basis of genetic differences and pathology, spotted fever group (SFG) and typhus group (TG). In China, 5 members of SFG have been identified in human cases ­­(*1*–*4*), and 7 kinds of *Rickettsia* have been detected from ticks or animals in the Qinghai–Tibet Plateau, including *R. heilongjiangensis, R. raoultii, R. slovaca,* and *R. sibirica*, which are known to be pathogenic to humans (*5*–*7*). However, clinical cases have not been reported. Thus, rickettsioses are probably neglected by local physicians and public health officers. We report a severe case of *R. sibirica* subspecies *sibirica* BJ-90 infection in this region.

A 50-year-old herdsman from Zhamashi, Qinghai Province, China, was hospitalized on July 13, 2018, because of intensive intermittent headache, anorexia, and chest tightness. On his fifth day of sheep shearing (designated as day 1), a blood-fed tick had been found on his head. The tick was removed by hand but its mouth parts remained in the man’s scalp. The next day, he became ill with fever, myalgia, itchiness, and asthenia. On day 5, his symptoms intensified and included severe intermittent headaches, which lasted for ≈10 minutes at each onset; high fever, up to 39.5°C; and fatigue, palpitation, nausea, and vomiting. Erythematous rashes appeared on his trunk, all 4 limbs, and the area behind the ears. Because signs of neurologic dysfunction, including confusion, drowsiness, and delirium appeared, he sought care at Qilian County Hospital on day 9, where he was treated for infectious endocarditis for 3 days before transfer to Qinghai State Hospital. During his visit at the Qinghai State Hospital, he was conscious and alert. Erythematous macules were observed over his trunk, elbow, and lower limbs. A 1.5 × 1.1 cm^2^ black eschar was visible at his right posterior occipital bone area; no tenderness was reported (Appendix Figure 1). The eschar was surgically excised on day 16. No lymphadenopathy was found. 

Alterations of the patient’s blood biochemistry included increased neutrophils (88.5% [reference 45%–75%]); decreased lymphocytes (9.3% [reference 20%–50%]), eosinophils (0% [reference 0.4%–8%]), and monocytes (1.9% [reference 3%–10%]); elevated creatine kinase–MB (42 U/L [reference 0–25 U/L]) and lactate dehydrogenase (445 U/L [reference 110–245 U/L]); and highly increased C-reactive protein (97.1 mg/dL [reference 0–5 mg/dL]), procalcitonin (0.433 ng/dL [reference 0–0.046 ng/dL]), D-dimers (12.28 μg/mL [reference 0–1.5 μg/mL]), fibrinogen degradation products (25 μg/mL [reference 0–5 μg/mL]), and β-microglobulin (4.5 μg/mL [reference 0.8–1.8 μg/mL]). The patient was prescribed levofloxacin lactate (0.5 g/d for 6 d). His symptoms subsided, and he was discharged on day 20.

On the basis of tick-bite history and the triad clinical characteristics (fever, rash, and eschar), nested PCR targeting the rickettsial citrate synthase conserved gene (*glt*A) was performed by using the eschar DNA as a template (Appendix). The 547-bp amplicon sequence shared 100% identity to *R. sibirica* 246, *R. sibirica* subsp. *sibirica* BJ-90, and *R. sibirica* subsp. *mongolitimonae* HA-91. The eschar DNA was sequenced by next-generation sequencing (BGI Genomics, https://www.bgi.com). A total of 21.6 Gb clean data were recovered from the high-throughput sequencing. Human reads (accounting for 99.9%) were filtered out. The remaining reads were mapped on the genome of *R. sibirica* 246 (GenBank accession no. AABW0100000). Rickettsial unique reads (n = 266) were analyzed against Refseq (https://www.ncbi.nlm.nih.gov/refseq; taxid 766). Most (226/266 [85%]) reads were 100% identical to *R. sibirica* subsp. *sibirica* BJ-90, whereas 213/266 (80%) were identical to *R. sibirica* 246, indicating that the *Rickettsia* was closest to *R. sibirica* subsp. *sibirica* BJ-90 (Table; Appendix Figure 2). Partial sequences of outer membrane protein A, outer membrane protein B, 17 kDa lipoprotein, surface cell antigen 1, and surface cell antigen 4 were amplified with specific primers. Phylogenetic trees showed that the Qinghai sequences clustered with *R. sibirica* subsp. *sibirica* BJ-90 (Figure; Appendix Figure 3). On the basis of next-generation sequencing data and PCR results, we concluded that the causative agent of the patient’s infection is closely related to *R. sibirica* subsp. *sibirica* BJ-90.

**Table:** Homology of 266 identified rickettsial unique reads shared by *Rickettsia* species, China*

% Identity	No. reads			
	BJ-90	*R. sibirica* 246†	HA-91‡	*R. heilongjiangensis*‡
100	226	213	153	48
99	25	35	76	65
98	6	9	19	46
1–97	9	9	17	102
0	0	0	1	5

*BJ-90, *R. sibirica* subsp. *sibirica* BJ-90; HA-91, *R. sibirica* subsp. *mongolitimonae* HA-91. †No significant difference (p = 0.35) compared with BJ-90 by Wilcoxon ranked nonparametric test. ‡Significant difference (p<0.05) compared with BJ-90 by Wilcoxon ranked nonparametric test.

**Figure:**
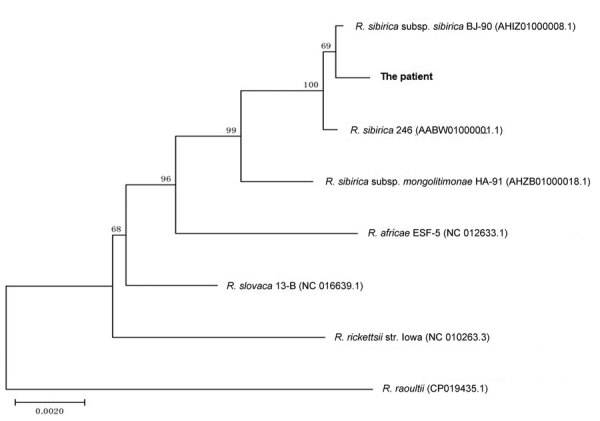
Phylogenetic analysis of concatenated nucleotide sequences from *Rickettsia* species collected in 2018 from eschar DNA from a patient in Qinghai Tibet Plateau, China (boldface), and reference sequences. A phylogenetic tree was constructed on the basis of the concatenated partial *glt*A, *omp*A, *omp*B, 17 kDa, *sca*1, and *sca*4 nucleotide sequences by using the neighbor-joining method with 1,000 bootstrap replicates. Numbers >70 indicate the bootstrapping value. GenBank accession numbers listed in Appendix Table 3 . Scale bar represents nucleotide substitutions.

We evaluated serum samples from the patient and persons from his surrounding community. Antibodies against *R. rickettsii* (SFG) and *R. typhi* (TG) were determined by indirect immunofluorescence assay. IgG titers of the patient’s paired serum samples on day 13 (1:128) and day 167 (1:4,096)) against SFG were increased by >4-fold, suggesting a recent infection with SFG. Approximately 17.4% (4/23) of the serum samples from the local community were positive for SFG, and 4.3% (1/23) were positive for TG (Appendix Table 1), indicating a high seroprevalence of SFG and co-circulation of TG in the region.

Because of the treating physicians’ unawareness of the prevalence of rickettsioses, the patient’s illness was misdiagnosed and incorrectly treated. In light of the fatal cases of *R. sibirica* subsp. *sibirica* infection recently documented in Russia and China (*8*–*10*), our report highlights the risk for rickettsial diseases among the public in the Qinghai–Tibet Plateau region and the urgent need for a large-scale seroepidemologic survey.

Appendix Additional information about severe case of rickettsiosis identified by metagenomic sequencing, China.
