# Supplementary material for: Severe Case of Rickettsiosis Identified by Metagenomic Sequencing, China
Source: Emerg Infect Dis. 2021 May;27(5):1530–2. doi: 10.3201/eid2705.203265 (PMC8084477; doi:10.3201/eid2705.203265)
Supplement: Appendix — Additional information about severe case of rickettsiosis identified by metagenomic sequencing, China. [file 20-3265-Techapp-s1.pdf]

Article DOI: https://doi.org/10.3201/eid2705.203265

Severe Case of Rickettsiosis Identified by Metagenomic Sequencing, China

Appendix

Materials and Methods

Ethics statements

Experimental protocols for collecting human clinical samples and isolating *Rickettsiae* from the samples were approved by the Ethical Review Committee of the National Institute for Communicable Disease Control and Prevention (ICDC), Chinese Center for Disease Control and Prevention (China CDC). Written consent regarding any potential identifiable images or data included in this article was obtained from all participants before the study.

DNA isolation and metagenomic sequencing

The eschar specimen (≈0.5 × 0.4 × 0.4 cm^3) was washed with PBS twice, shredded, and homogenized with TissueLyser II (QIAGEN, USA) in up to 1 ml of PBS. Homogenate DNA (200 µL) was extracted (QIAamp Tissue kit, QIAGEN, USA). DNA aliquot were stocked at −80°C and 4°C until use. Quality and quantity of the DNA were evaluated by Nanodrop spectrophotometer (Thermo Scientific, USA).

A library of eschar DNA (2 µg) was constructed (BGI Genomics, China) and sequenced using BGIseq2000. The dataset was cleaned by removing the adaptors and low quality reads. Reads mapped to the hg38 genome were filtered out. The remaining reads were mapped to the reference genome of *R. sibirica strain* 246 using bowtie2 with default parameters. Sequence of the individual rickettsial reads was analyzed by blastn against NCBI Refseq Genome Database of *Rickettesias* (taxid:766). A phylogenetic tree was constructed using iqtree software on the basis

of the sequence of the concatenated rickettsial unique reads and their homologous segments in seven complete genomes of *Rickettsia.*

Molecular diagnosis and amplicon sequencing

Primers targeting rickettsial species-specific genes of citrate synthase (*glt*A) and outer membrane protein A (*omp*A), outer membrane protein B (*omp*B), 17kDa lipoprotein (17kDa), surface cell antigen1 (*sca*1), and surface cell antigen 4 (*sac*4) were verified using chromosomal DNA of *R. rickettsii* (Appendix Table 1). To avoid cross-contamination, positive controls were skipped when field samples were screened. Eschar DNA (200 ng/reaction) was used as template for PCR under condition of 95°C for 5 minutes (one cycle), 95°C for 20 seconds, 51–53°C for 20 seconds, 68°C for 30 seconds (30 cycles), followed by 68°C for 5 minutes. When PCR band was faint on agarose gel (loading 5 µL of PCR product), a second PCR was performed as described above using the original PCR product (2 µl) as template. The high fidelity PCR products were Sanger sequenced and used for blastn search. Phylogenetic trees were generated by the neighbor-joining method implemented in MEGA 6.06 software with bootstrap replicates of 1000.

Serologic test

The first blood sample was collected when the patient was admitted at the Qinghai State Hospital on day 13. A follow-up sample was obtained about 5 months after his release from hospital (on day 167). Serum samples from the surrounding community were collected (male = 6, female = 17; ages 6–75 years old). All sera were examined for IgG and IgM against SFGR (*R. rickettsii)* and TG (*R. typhi)* by indirect immunofluorescence assay (IFA) following the manufacturer's instructions (Focus Diagnostic Inc. Cypress, USA). The sera were 2-fold serial diluted starting at 1:2 and initially tested at dilution of 1:32 antibody titers were further determined for the blood samples that demonstrated strong fluorescence at the primary screening. A titer was defined as reciprocal of the last dilution that specific fluorescence can be observed (comparing with positive and negative control). A titer of ≥64 was designated as a positive exposure history of SFGR. A recent infection was considered if the titers of a person's paired serum samples, collected in 2 weeks apart, increased by ≥4-fold (Appendix Table 2).

Statistical analyses

Identities (%) of the 266 rickettsial reads matched to *R. sibirica* subsp. *sibirica BJ-90, R. sibirica 246, R. sibirica subsp. mongolitimonae* HA-91, and *R. helongjiangensis* were analyzed using Wilcoxon ranked nonparametric test by SPSS 21.0 software (SPSS Inc., Chicago, IL, USA). A paired Student's *t*-test was performed using Microsoft Excel for statistical difference determination (p-value ≤0.05).

Nucleotide sequence accession number

Nucleotide sequences obtained in this study were deposited in the GenBank and the National Genomic Data Center (NGDC) (Appendix Table 3).

Reference

1. Sekeyova Z, Roux V, Raoult D. Phylogeny of *Rickettsia* spp. inferred by comparing sequences of 'gene D', which encodes an intracytoplasmic protein. Int J Syst Evol Microbiol. 2001;51:1353–60. PubMed https://doi.org/10.1099/00207713-51-4-1353

Appendix Table 1. Titer of IgG to *R. rickettsii* and *R. typhi* among the local population in Zhamashi, Qilian county, Qinghai province*

Sample no.	Gender	Age	*R. rickettsia*	*R. typhus*
1	M	68	<32	<32
2	M	51	<32	<32
3	F	42	<32	<32
4	F	48	<32	<32
5	F	44	= 128	<32
6	M	6	<32	<32
7	F	37	= 128	<32
8	F	57	<32	<32
9	F	75	<32	= 128
10	M	64	<32	<32
11	F	71	<32	<32
12	F	61	<32	<32
13	F	70	<32	<32
14	F	27	<32	<32
15	F	26	<32	<32
16	F	30	= 64	<32
17	M	40	= 64	<32
18	M	61	<32	<32
19	F	46	<32	<32
20	F	72	<32	<32
21	F	58	<32	<32
22	F	50	<32	<32
23	F	42	<32	<32
The patient†	M	50	= 128	<32
The patient‡	M	50	= 4096	<32

*Blood samples (n = 23) were collected in the patient’s local area, including 6 males and 17 females, ages 6–72, and determined by indirect immunofluorensence assay. Positive was defined when titer of IgG was ≥1:64.

†The patient's serum on day 13.

‡The patient's serum on day 167.

Appendix Table 2. Primers for PCR and sequencing

Primer	Target gene	Sequence (5′-3′)	Reference
R001F	*glt*A	ttgtcagtctactatcaccTATATA	This study
R001R		gaagcattctgctcatgatcggcat	This study
R002F	*omp*A	GGAGTAATGTAGCAGGTACCGCTAG	This study
R002R		ccgcccaatattaccggtttgag	This study
R005F	*omp*B	GTAACCGGAAGTAATCGTTTCGTAAA	This study
R005R		CTTTATAACCAGCTAAACCACCTT	This study
R008F	*sca*1	GAGAGTCTCATATAAAAAGAGG	This study
R008R		ATGTTCTACCGCTCCTTGG	This study
R010F	17kDa	GCTCTTGCAACTTCTATGTTACA	This study
R010R		CATTGTTCGTCAGGTTGGCGgCATG	This study
R011F	*sca*4	atgagtaaagacggtaacct	(*1*)
R011R		aagctattgcgtcatctccg	(*1*)

Appendix Table 3. Information of nucleotide sequences obtained in this study and GenBank accession numbers*

		Identity to (%)			
Targeting gene†	Amplicon size (bp)	BJ-90	246	HA-91	GenBank accession no.
*glt*A	547	100	100	100	MT521745
*omp*A	619	99.51	99.51	98.68	MT521746
*omp*B	512	99.80	99.41	99.02	MT521747
*sca*1	648	99.84	99.84	99.22	MT521748
17kDa	441	99.77	99.77	99.54	MT521749
*sca*4	931	99.24	99.13	98.36	MT521750
Reads‡	21600§	99.7	99.63	98.9	CRA002872¶

*BJ-90, *R. sibirica* subsp. *sibirica* BJ-90; 246, R. sibirica 246; HA-91, *R. sibirica* subsp. *mongolitimonae* HA-91.

†*glt*A, citrate synthase; *omp*A, outer membrane protein A; *omp*B, outer membrane protein B; 17kDa, 17kDa lipoprotein; *sca*1, surface cell antigen 1; *sac*4, surface cell antigen 4.

‡Reads of metagenomic sequencing.

§Total nucleotides of the rickettsial reads (266 reads, 100bp/read).

¶Accession number of the 21.6Gb metagenomic sequencing which were deposited in the National Genomic Data Center (NGDC).

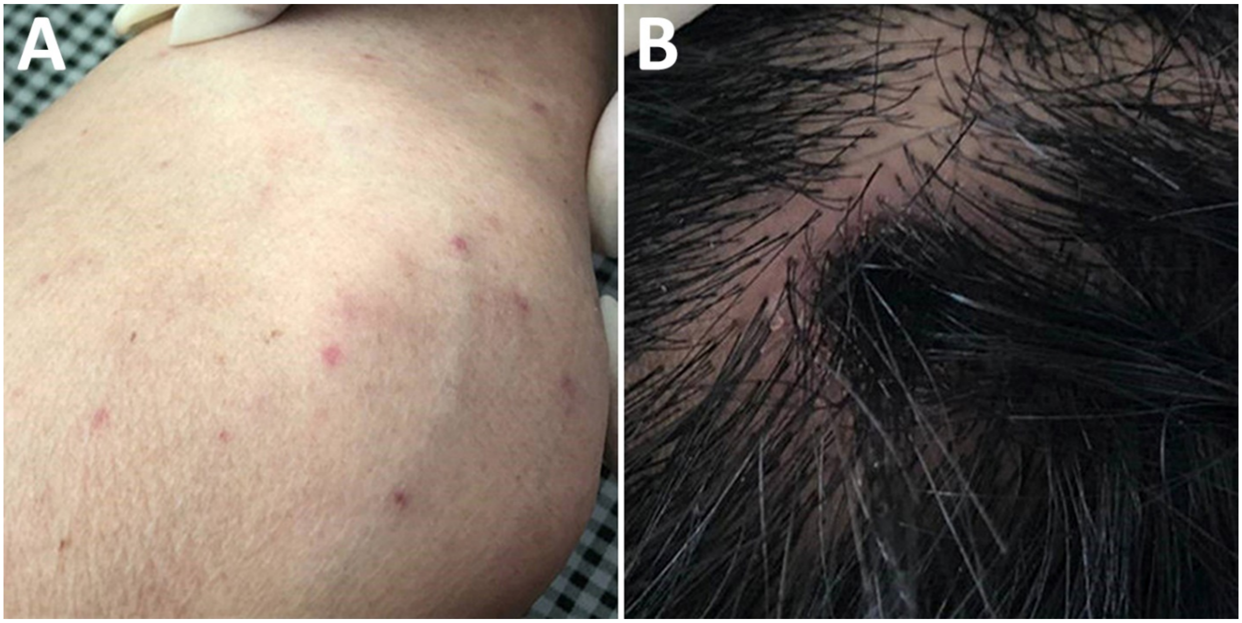

Appendix Figure 1. Macular rash on the right knee area of the patient on 5 day (A) and the eschar at right posterior occipital region on day 16 (B) after recognition of a tick bite.

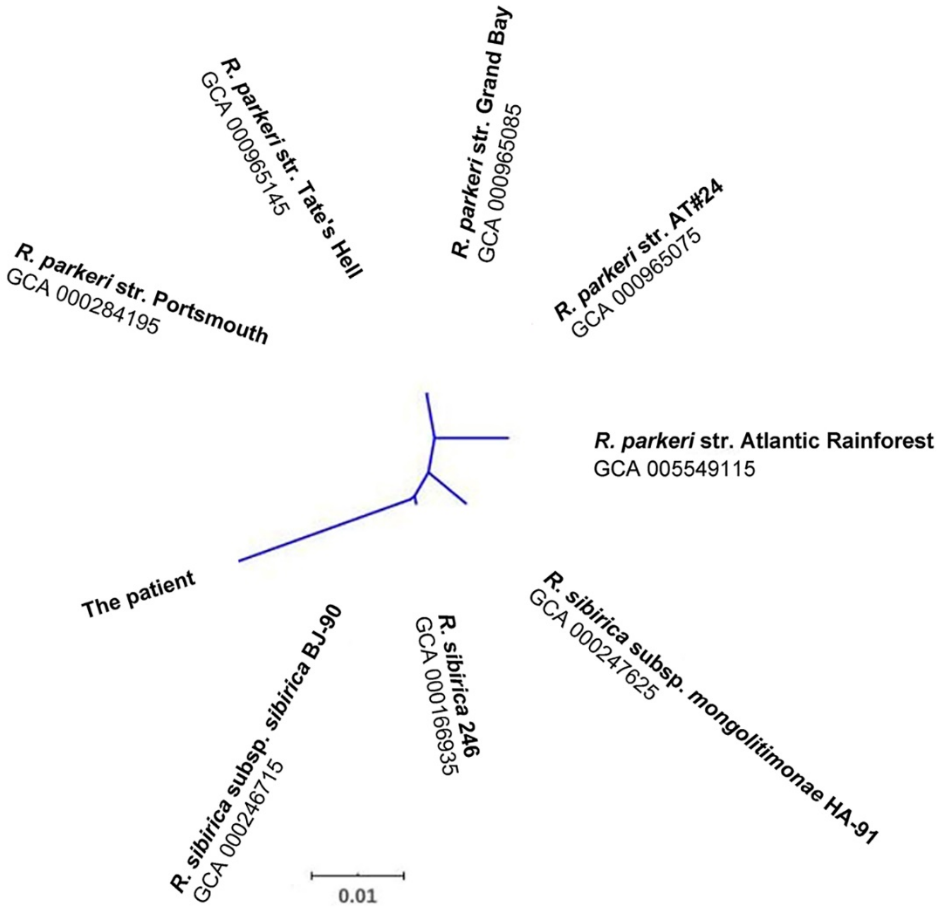

Appendix Figure 2. The causative agent of the Qinghai patient is closely related to *R.sibirica* subsp. *sibirica* BJ-90. An unrooted phylogenetic tree was generated on the basis of the concatenated rickettsial reads obtained from metagenomic sequencing of eschar DNA. Scale bar represents nucleotide distance.

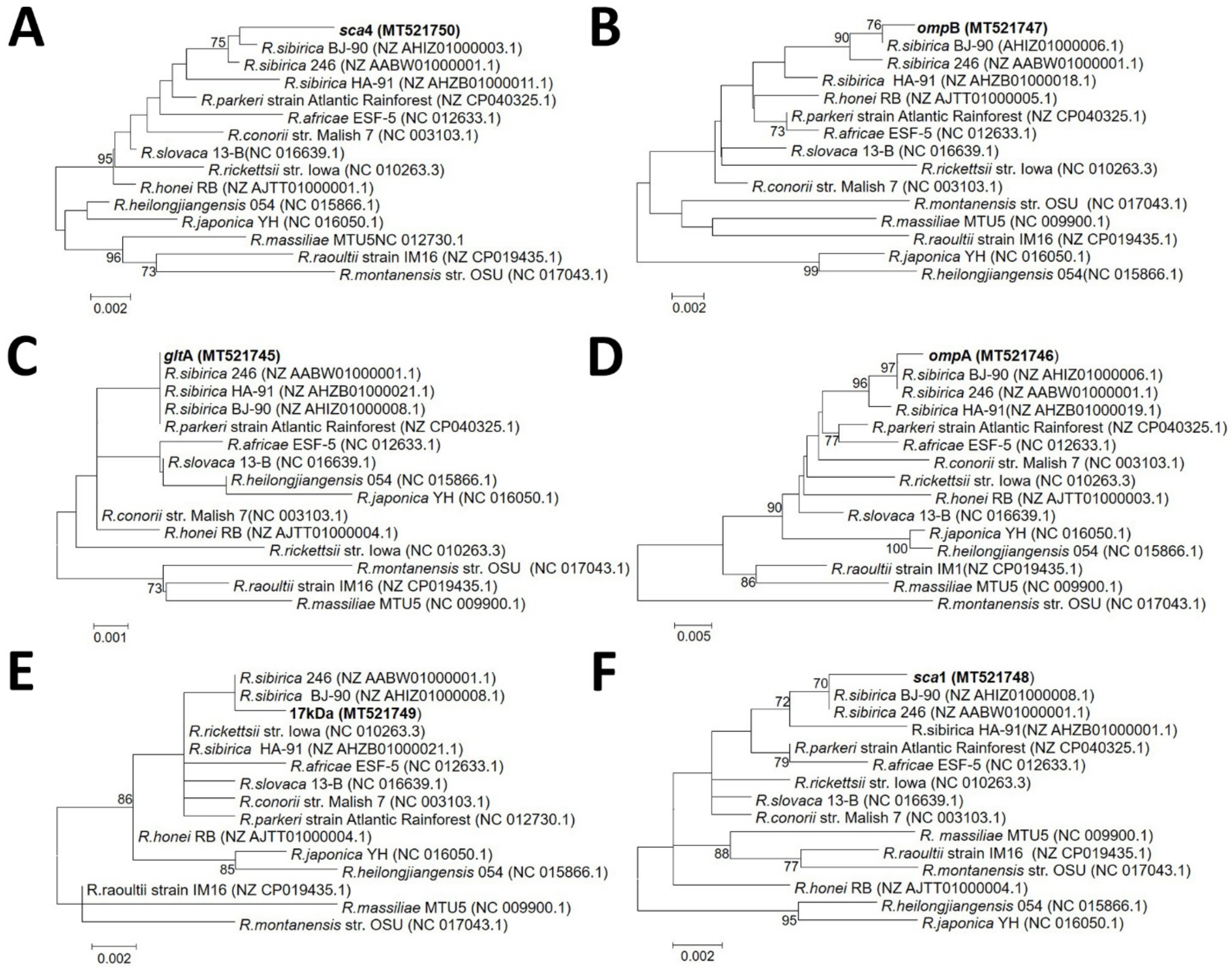

Appendix Figure 3. Phylogenetic analysis of the causative agent. Phylogenetic tree was constructed on the basis of sequences of partial *glt*A, *omp*A, *omp*B,17kDa, *sca*1, and *sca*4. Amplicon sequences were aligned using MUSCLE within the MEGA 6 software. Phylogenetic relationships were analyzed using the neighbor-joining method with 1000 bootstrap replicates. Values >70 were indicated at the nodes. Bold represents the sequences obtained in this study. Scale bar indicates number of nucleotide substitutions.
